# TaNRAMP3 is essential for manganese transport in *Triticum aestivum*

**DOI:** 10.1007/s44154-023-00120-2

**Published:** 2023-09-22

**Authors:** Zhangqing Wang, Yanting Zhang, Chenyu Cao, Jiaming Liu, Yuan Deng, Zhenqian Zhang, Cun Wang

**Affiliations:** National Key Laboratory of Crop Improvement for Stress Tolerance and Production, Northwest A&F University, Yangling, Shaanxi 712100 People’s Republic of China

**Keywords:** Manganese, *Triticum aestivum*, TaNRAMP3, Transporter

## Abstract

Manganese (Mn) is an essential trace element for almost all living organisms. In plants, Mn deficiency, which is occurs in calcareous soils or alkaline soils, severely limiting crop yields. However, the potential mechanism of Mn transport in *Triticum aestivum* is still obscure. Here, we found that TaNRAMP3, a member of the naturally resistant macrophage protein (NRAMP) family in *Triticum aestivum*, is located in the plasma membrane of protoplasts and functions as an influx transporter for Mn in yeast (Δ*smf1*). The expression of *TaNRAMP3* was induced under Mn-deficiency conditions. Furthermore, *TaNRAMP3-RNAi* plants exhibited a sensitive phenotype, while transgenic plants overexpressing *TaNRAMP3* showed a tolerant phenotype. In addition, TaNRAMP3 rescued the sensitive phenotype of Arabidopsis* nramp1* mutant under Mn deficiency condition. In summary, our study reveals the key role of TaNRAMP3 in Mn transport in *Triticum aestivum*, allowing it to adapt to Mn-deficiency stress. These findings provide new insights for the cultivation of Mn-deficiency tolerant wheat varieties.

**Supplementary Information:**

The online version contains supplementary material available at 10.1007/s44154-023-00120-2.

## Introduction

Manganese (Mn) is an essential micronutrient for plant growth and an important regulatory factor for various cellular processes, maintaining metabolic functions in different plant cell compartments (Li et al. 2019). In plants, Mn participates in many important metabolic processes, especially the indispensable catalytic role in Photosystem II (PSII) (Alscher et al. 2002; Schmidt and Husted 2019; Socha and Guerinot 2014; Xie et al. 2023). Mn deficiency in plants is a serious and widespread plant nutritional disorder that particularly prevalent in alkaline soils, which comprise 30% of arable land, due to conditions that favor the oxidation of Mn to unavailable Mn oxides (Alejandro et al. 2020; Schmidt et al. 2016).

The Mn homeostasis in plants is formed by complex interactions between root absorption, organ distribution, and allocation. The acquisition and long-distance transport of Mn are mediated by members of various Mn transporter protein families, such as natural resistance related macrophage proteins (NRAMP) (Cailliatte et al. 2010; Castaings et al. 2016), cation diffusion promoters/metal tolerance proteins (CDF/MTP) (Delhaize et al. 2007; Eroglu et al. 2016), and so forth. The Mn transporters possess different subcellular localization and functions, which are crucial for maintaining intracellular Mn homeostasis. In addition, the activities and locations of these Mn transporters are strictly and precise regulated by Ca^2+^ signaling at the translation level (Zhang et al. 2021b, 2023a; Fu et al. 2022; Ju et al. 2022).

Six NRAMP transporter families play important roles in ion homeostasis in Arabidopsis (Alejandro et al. 2017; Eisenhut et al. 2018). AtNRAMP1, a major Mn transporter located in the plasma membrane (PM) of root epidermis and cortical cells, mediates the entry of Mn into roots (Cailliatte et al. 2010; Li et al. 2022). AtNRAMP2 is located in the Golgi apparatus network and is responsible for the distribution of Mn within the cell and for maintaining cellular redox homeostasis in the absence of Mn (Alejandro et al. 2017). AtNRAMP3 and AtNRAMP4 are located on the vacuole membrane and are responsible for releasing Mn or Fe from the vacuole (Lanquar et al. 2005, 2010). AtNRAMP6, located on the PM, plays an important role in the detoxification of cadmium (Cd) (Cailliatte et al. 2009; Zhang et al. 2023b). In rice, OsNRAMP5 is located at the far end of the outer and endodermis cells, and is the main transporter of Mn and Cd, responsible for transporting Mn and Cd from the external solution to the root cells (Sasaki et al. 2012; Ueno et al. 2015). Recent studies have reported that ZmNRAMP2 in maize is located on tonoplast to mediate Mn transport (Guo et al. 2022).

However, the physiological contribution of Mn transport in the NRAMP family in *Triticum aestivum* is still unclear. In this study, subcellular localization analysis demonstrated that TaNRAMP3 was located in the PM. The Mn transport function of TaNRAMP3 is verified through a heterologous yeast complementation test. By using knockdown mutants and transgenic overexpressed lines, we demonstrated the role of TaNRAMP3 in Mn transport, which is essential for maintaining plant photosynthetic activity and growth under Mn-deficiency conditions.

## Results

### TaNRAMP3 encodes a plasma membrane localization protein

Since Mn transport is mainly mediated by the NRAMP family in Arabidopsis, we performed the protein sequences alignment between the NRAMP family members of Arabidopsis and *Triticum aestivum*. The seven NRAMP family members of *Triticum aestivum* were obtained on the website (https://plants.ensembl.org) were TaNRAMP1 (TraesCS7A02G327300), TaNRAMP2 (TraesCS4A02G050500) and TaNRAMP3 (TraesCS7A02G464300), TaNRAMP5 (TraesCS4A02G004400), TaNRAMP4 (TraesCSU02G077000), TaNRAMP6 (TraesCS3A02G195100), TaNRAMP7 (TraesCS5A02G072200). The results of the phylogenetic analysis showed that TaNRAMP3 is the closest homolog of AtNRAMP1, indicating that TaNRAMP3 and AtNRAMP1 may have similar transport roles (Supplemental Figure 1).

**Fig. 1:**
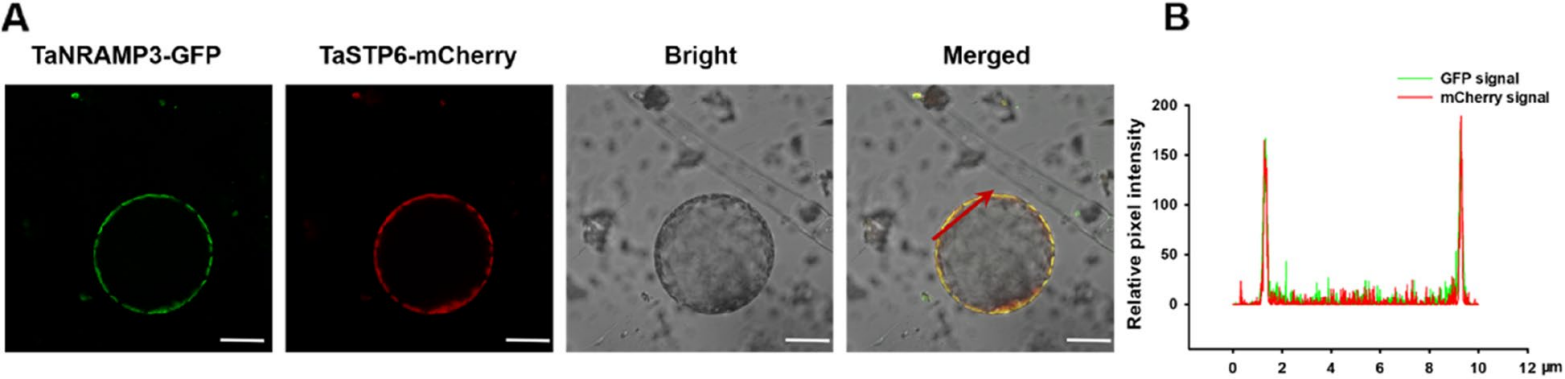
Subcellular localization of TaNRAMP3 in *Triticum aestivum*. **A** Plasma membrane localization of TaNRAMP3 in *Triticum aestivum* mesophyll protoplasts. TaNRAMP3 is fused to GFP. The PM marker is TaSTP6-mCherry. Scale bars, 5 μm. **B** Fluorescence analysis of the localization of the interaction between TaNRAMP3-GFP and TaSTP6-mCherry in *Triticum aestivum* mesophyll protoplasts. The fluorescence intensity (mCherry and GFP signals) of the section lines was scanned using Fiji/ImageJ software. The location of the section line is marked red arrow in (**A**)

The CDS of TaNRAMP3 is predicted to have 1,647 nucleotide residues and encode a 548-amino acid peptide with a calculated molecular mass of 59.7 kD. Analysis of the membrane spanning model indicated that *TaNRAMP3* contains 12 predicted transmembrane domains (Supplemental Figure 2). In order to investigate the subcellular localization of TaNRAMP3, we fused the GFP tag to *TaNRAMP3* and co-expressed it with TaSP6-mCherry (a PM marker) (Huai et al. 2019) in *Triticum aestivum* protoplasts. The results showed that TaNRAMP3 was localized in the PM (Fig. 1A). To confirm the overlap of TaNRAMP3 with TaSP6-mCherry at the microscopic level, we analyzed the fluorescence signals using Fiji/ImageJ and observed that the fluorescence peaks of the GFP signal and mCherry signal overlapped (Fig. 1B).

### TaNRAMP3 protein mediates Mn transport in yeast

**Fig. 2:**
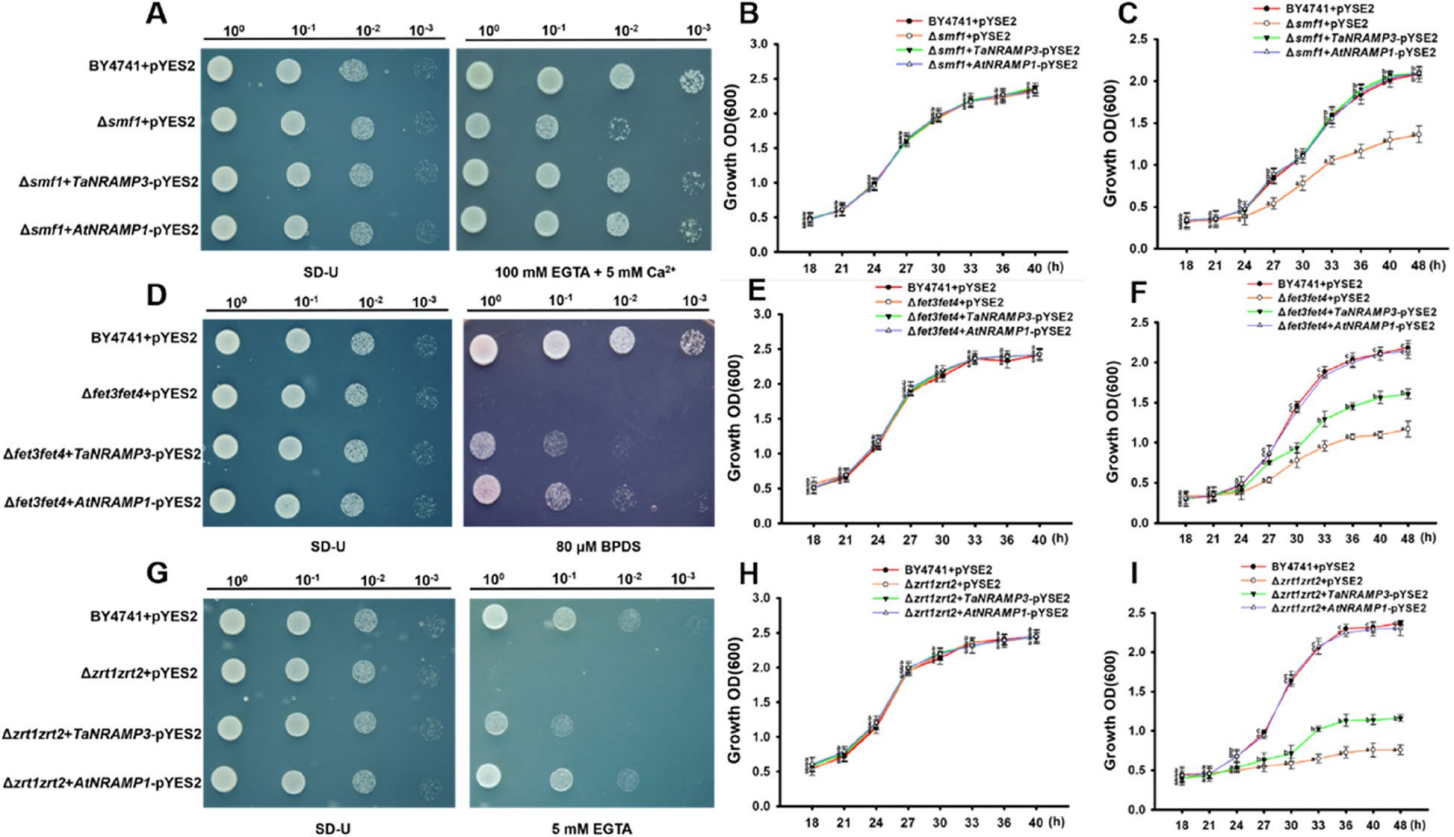
Functional analysis of TaNRAMP3 on ion transport in yeast. **A** Potential function analysis of TaNRAMP3 on Mn transport. Growth of yeast strains on normal medium (SD-U, lacking Ura) and treatment medium (SD-U + 100 mM EGTA + 5 mM Ca^2+^) for 3–5 d. Four 10-fold series of diluents were established under sterile conditions. **B** Growth curves of yeast cells were plotted against OD600 values in normal SD-U nutrient solution. **C** Growth curves of yeast cells were plotted against OD600 values in SD-U nutrient solution containing 2 mM EGTA. **D** Potential function analysis of TaNRAMP3 on Mn transport. Growth of yeast strains on normal medium (SD-U, lacking Ura) and treatment medium (SD-U + 80 μM BPDS) for 3–5 d. Four 10-fold series of diluents were established under sterile conditions. **E** Growth curves of yeast cells were plotted against OD600 values in normal SD-U nutrient solution. **F** Growth curves of yeast cells were plotted against OD600 values in SD-U nutrient solution containing 20 μM BPDS. **G** Potential function analysis of TaNRAMP3 on Mn transport. Growth of yeast strains on normal medium (SD-U, lacking Ura) and treatment medium (SD-U + 5 mM EGTA) for 3–5 d. Four 10-fold series of diluents were established under sterile conditions. **H** Growth curves of yeast cells were plotted against OD600 values in normal SD-U nutrient solution. **I** Growth curves of yeast cells were plotted against OD600 values in SD-U nutrient solution containing 1 mM EGTA

Since TaNRAMP3 and AtNRAMP1 share a high degree of sequence identity, and are localized to the PM, we speculate that TaNRAMP3 fulfill the metal ion transport activity, particularly for Mn ions. To elucidate the transport function of TaNRAMP3 via heterologous yeast complementing analysis, the full-length CDS of TaNRAMP3 was cloned into pYES2 vector, and then transformed into wild type yeast (BY4741), yeast mutant *Δsmf1* (a low Mn sensitive yeast mutant) (Zhang et al. 2018, 2021a), *Δfet3fet4* (a low Fe sensitive yeast mutant) (Khan et al. 2019), and *Δzrt1zrt2* (a low Zn sensitive yeast mutant) (Lee et al. 2021). The deficient environment of elements (Mn, Fe, and Zn) was created by adding an appropriate amount of EGTA (divalent cationic chelating agent) or BPDS (chelated iron ion), and the growth of yeast was detected. AtNRAMP1 was used as a positive control. The results showed that yeast expressing TaNRAMP3 could rescue the *Δsmf1* sensitive state under low Mn condition, while empty vector could not complement (Fig. 2A). Growth curve analysis showed that the yeast expressing TaNRAMP3 and AtNRAMP1 exhibited the similar growth trend, and both could restore the sensitive state of *Δsmf1* to wild-type state (Fig. 2C). This indicated that TaNRAMP3 functions as an influx transporter for Mn.

However, yeast expressing TaNRAMP3 can only partially restore the sensitive phenotype of *Δfet3fet4* and *Δzrt1zrt2* compared to wild-type yeast, and growth curve analysis indicated that yeast expressing TaNRAMP3 has limited growth under Fe or Zn deficiency conditions (Fig. 2D-I). This indicated that TaNRAMP3 has a weaker ability to transport Fe and Zn compared to AtNRAMP1, and its transport capacity is relatively weak. To confirm that the differences in Fe and Zn transport by TaNRAMP3 were not due to differences in its expression levels in yeast, semi-qRT-PCR was performed, and the results showed similar expression levels of *TaNRAMP3* and *AtNRAMP1* in each yeast mutant (Supplemental Figure 3). This indicates that there is indeed a difference in transport between TaNRAMP3 and AtNRAMP1, which is not caused by variations in expression levels.

To explore the spatiotemporal expression patterns of TaNRAMP3, different tissues of wild type *Triticum aestivum* were selected for qRT-PCR analysis under conditions of Mn sufficiency and Mn depletion. The results showed that *TaNRAMP3* was expressed not only in the roots but also in the leaves, with its expression level in the leaves being 7–11 times higher than that in the roots. Furthermore, we observed that the expression levels of *TaNRAMP3* were increased approximately 2 times in the roots and leaves under Mn deficiency condition (Supplemental Figure 4).

### TaNRAMP3 enhances plants tolerance to Mn deficiency

**Fig. 3:**
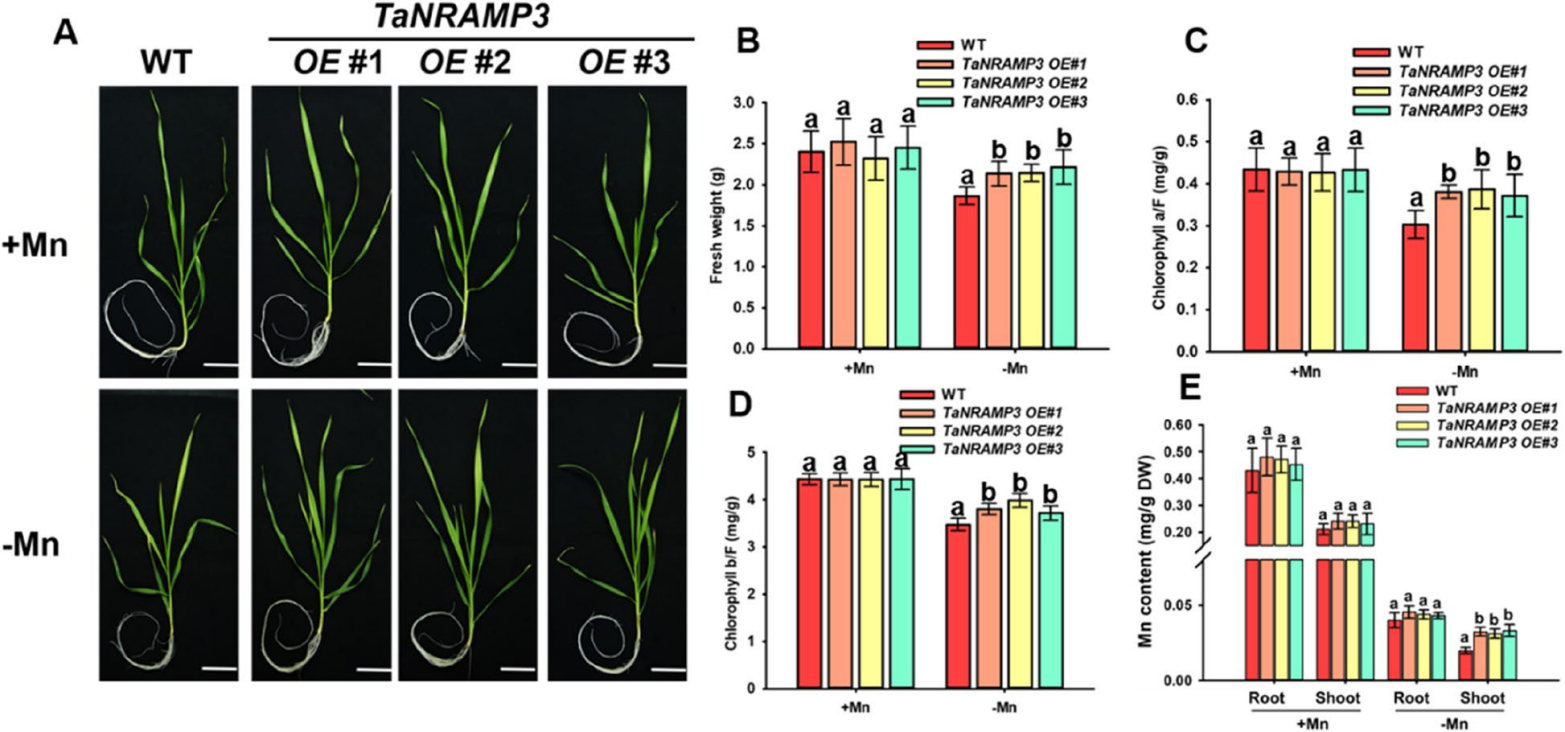
Phenotypic analysis of overexpressed plants. **A** Mn deficiency phenotypes of TaNRAMP3-OE plants. Scale bars, 5 cm. **B** Statistical analysis of fresh weight of plants shown in (**A**). **C** Chlorophyll a content analysis of TaNRAMP3-OE and WT plants shown in (**A**). The results are presented as the mean ± SD (*n* = 4 biological replicates, 4 shoots). Statistical differences were calculated by one-way ANOVA. Different letters indicate means that were statistically different by Tukey’s multiple testing method (*P* < 0.05) for genotypes within a given growth condition (+ Mn or -Mn). **D** Chlorophyll b content analysis of TaNRAMP3-OE and WT plants shown in (**A**). The results are presented as the mean ± SD (*n* = 4 biological replicates, 4 shoots). Statistical differences were calculated by one-way ANOVA. Different letters indicate means that were statistically different by Tukey’s multiple testing method (*P* < 0.05) for genotypes within a given growth condition (+ Mn or -Mn). **E** Mn content analysis of the TaNRAMP3-OE and WT plants. The results are presented as the mean ± SD (*n* = 3 biological replicates, 9 roots or 9 shoots). Statistical differences were calculated by one-way ANOVA. Different letters indicate means that were statistically different by Tukey’s multiple testing method (*P* < 0.05) for genotypes within a given growth condition (+ Mn or -Mn)

**Fig. 4:**
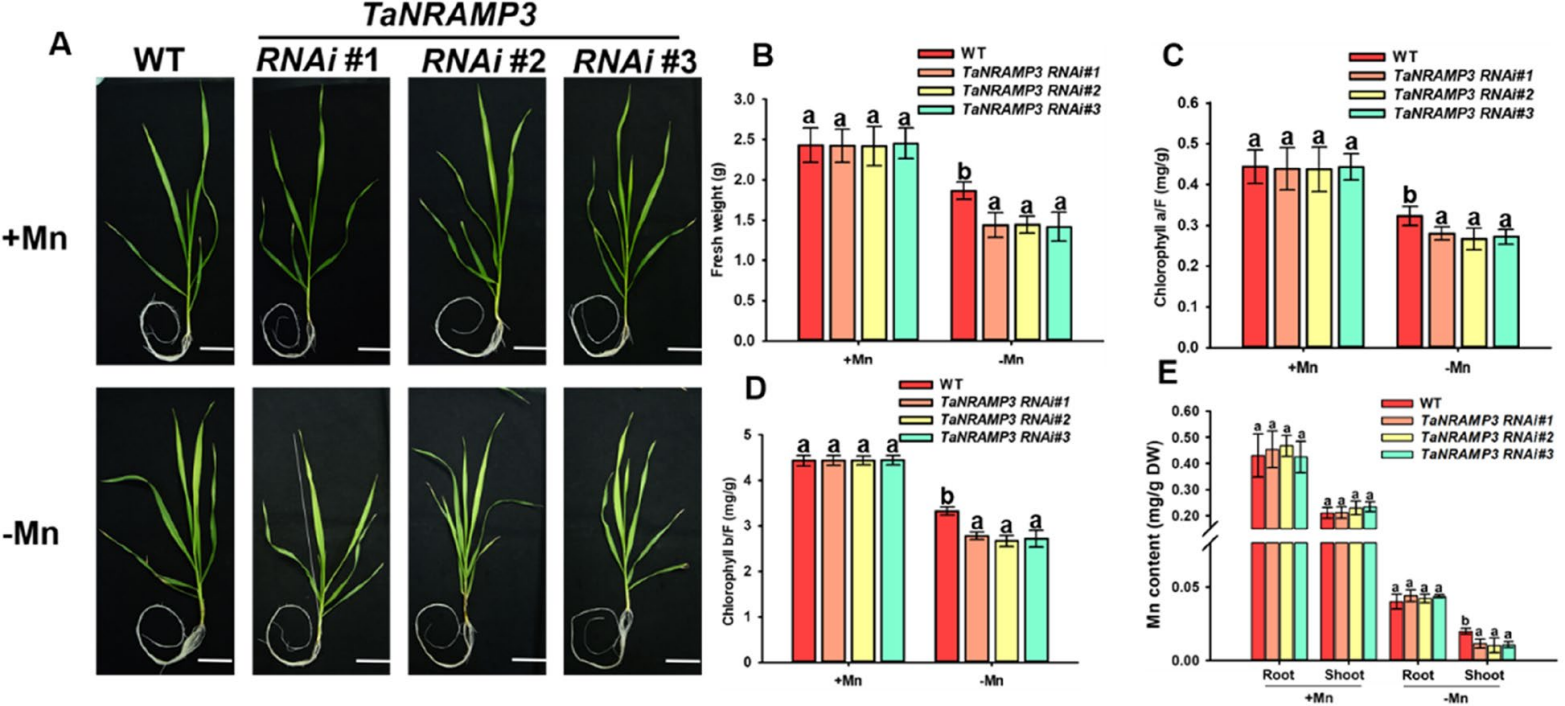
Phenotypic analysis of *TaNRAMP3-RNAi* plants. **A** Mn deficiency phenotypes of *TaNRAMP3-RNAi* plants. Scale bars, 5 cm. **B** Statistical analysis of fresh weight of plants shown in (**A**). **C** Chlorophyll a content analysis of *TaNRAMP3-RNAi* and WT plants shown in (**A**). The results are presented as the mean ± SD (*n* = 4 biological replicates, 4 shoots). Statistical differences were calculated by one-way ANOVA. Different letters indicate means that were statistically different by Tukey’s multiple testing method (*P* < 0.05) for genotypes within a given growth condition (+ Mn or -Mn). **D** Chlorophyll b content analysis of TaNRAMP3-OE and WT plants shown in (**A**). The results are presented as the mean ± SD (*n* = 4 biological replicates, 4 shoots). Statistical differences were calculated by one-way ANOVA. Different letters indicate means that were statistically different by Tukey’s multiple testing method (*P* < 0.05) for genotypes within a given growth condition (+ Mn or -Mn). **E** Mn content analysis of the TaNRAMP3-OE and WT plants. The results are presented as the mean ± SD (*n* = 3 biological replicates, 9 roots or 9 shoots). Statistical differences were calculated by one-way ANOVA. Different letters indicate means that were statistically different by Tukey’s multiple testing method (*P* < 0.05) for genotypes within a given growth condition (+ Mn or -Mn)

To investigate the potential role of TaNRAMP3 in mediating Mn transport in plants, we generated transgenic lines of *Triticum aestivum* overexpressing *TaNRAMP3* (*TaNRAMP3-OE*) and lines with RNA-interference-mediated downregulation of *TaNRAMP3* (*TaNRAMP3-RNAi*) through Agrobacterium-mediated transformation. Three lines of *TaNRAMP3-OE* and three lines of *TaNRAMP3-RNAi* plants were selected for further analysis. Compared to the WT, the *TaNRAMP3 e*xpression levels were approximately 2-fold higher in the *TaNRAMP3-OE* lines, and approximately 5-fold lower in the *TaNRAMP3-RNAi* lines (Supplemental Figure 5). To verify whether RNA interference of *TaNRAMP3* affect the expression levels of other *TaNRAMPs* (*TaNRAMP1*, *TaNRAMP2*, *TaNRAMP4*, *TaNRAMP5*, *TaNRAMP6*, *TaNRAMP7*), we conducted expression analysis of other *TaNRAMPs* in both wild-type (WT) and *TaNRAMP3-RNAi* plants. Interestingly, the results indicated that there is no significant difference in the expression levels of other *TaNRAMPs* between *TaNRAMP3-RNAi* plants and the WT plants (Supplemental Figure 6). These findings suggest that the down-regulation observed in *TaNRAMP3-RNAi* plants specifically affects *TaNRAMP3* expression, without impacting the expression of other *TaNRAMPs*. The hydroponic phenotypes revealed that *TaNRAMP3-OE* plants exhibited increased vigor and greener leaves compared to WT plants under Mn-deficiency conditions. To further investigate the impact of *TaNRAMP3* on plant growth, we measured the fresh weight and chlorophyll content of *TaNRAMP3-OE* transgenic plants. The results demonstrated that both fresh weight and chlorophyll content were higher in *TaNRAMP3-OE* plants compared to WT plants (Fig. 3A-D). In contrast, *TaNRAMP3-RNAi* plants displayed leaf chlorosis and subsequent inhibition of plant growth and development, resulting in decreased fresh weight and chlorophyll content compared to WT plants (Fig. 4A-D). Under normal growth conditions, no significant differences were observed among the *TaNRAMP3-OE* plants, *TaNRAMP3-RNAi* plants, and WT plants.

To determine whether the low Mn phenotypes observed in *TaNRAMP3-OE* and *TaNRAMP3-RNAi* plants are a result of changes in Mn content, we determined the Mn concentration of WT, *TaNRAMP3-OE* and *TaNRAMP3-RNAi* plants under hydroponic culture. When subjected to Mn deficiency, we observed a significant increase in Mn content in the shoots of *TaNRAMP3-OE* plants, while the Mn content in the shoots of *TaNRAMP3-RNAi* plants showed a decrease compared to WT plants (Figs. 3E and 4E). Conversely, under Mn-sufficient conditions, there were no significant differences in Mn content between *TaNRAMP3-OE*, *TaNRAMP3-RNAi*, and WT plants in either the roots or shoots. These findings further support the notion that TaNRAMP3 plays a crucial role in regulating Mn homeostasis in plants.

### *TaNRAMP3* is capable of restoring the sensitive phenotype of *nramp1* mutant under Mn deficiency in Arabidopsis

**Fig. 5:**
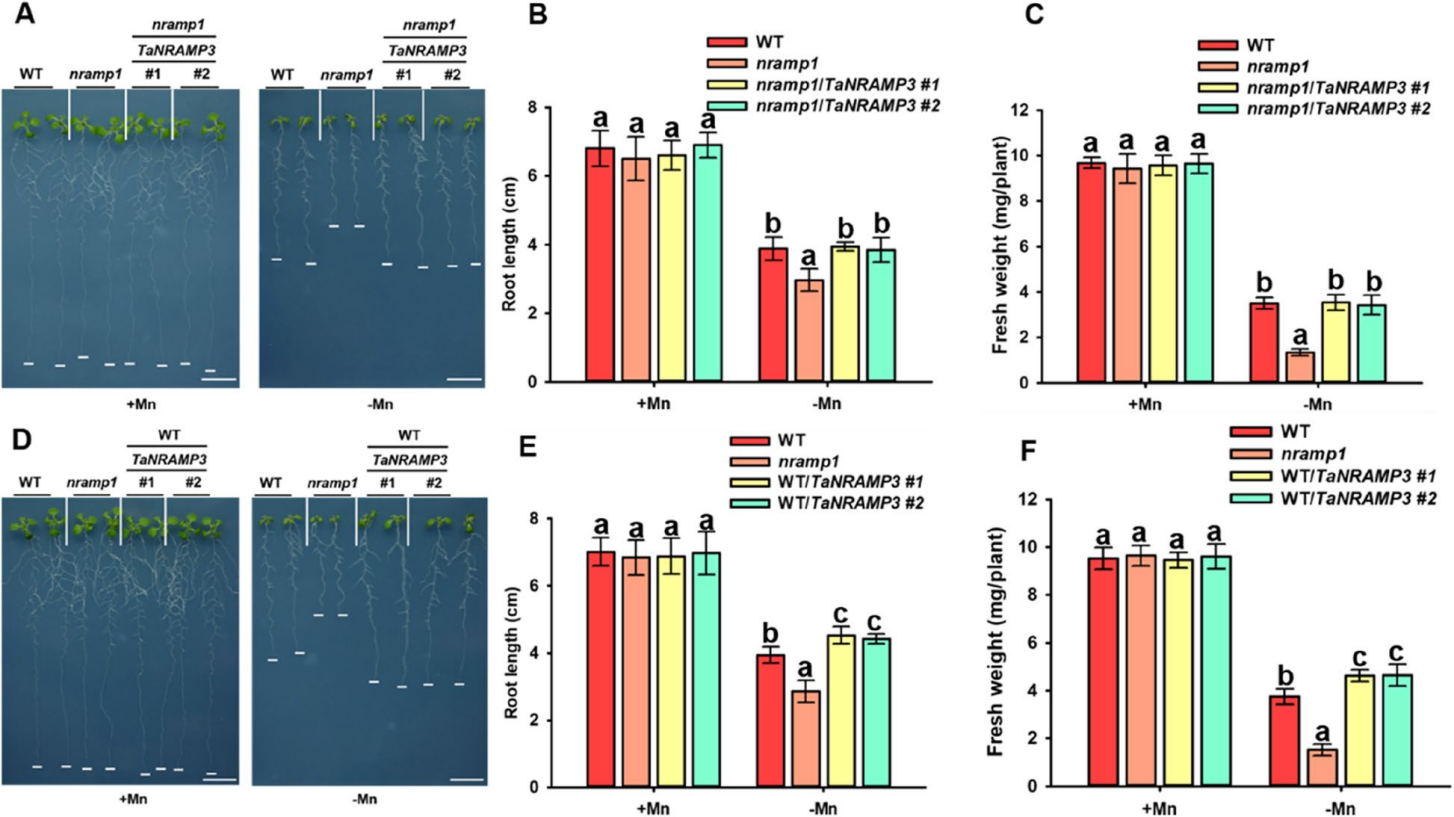
Potential functional analysis of AtNRAMP1 and TaNRAMP3 in Arabidopsis. **A** Mn deficiency phenotypes of WT, *nramp1* and *nramp1*/*TaNRAMP3* transgenic plants. Scale bars, 1 cm. **B** Statistical analysis of root lengths of plants shown in (**A**). The data are presented as the mean ± SD (*n* = 15 seedlings for each genotype). **C** Statistical analysis of fresh weight of plants shown in (**A**). The data are presented as the mean ± SD (*n* = 15 seedlings for each genotype). **D** Mn deficiency phenotypes of WT, *nramp1* and WT/*TaNRAMP3* transgenic plants. Scale bars, 1 cm. **E** Statistical analysis of root lengths of plants shown in (**A**). The data are presented as the mean ± SD (*n* = 15 seedlings for each genotype). **F** Statistical analysis of fresh weight of plants shown in (**A**). The data are presented as the mean ± SD (*n* = 15 seedlings for each genotype)

Since the distribution of Mn content in *Triticum aestivum*, which is mediated by TaNRAMP3 under Mn deficiency condition, is similar to that mediated by NRAMP1 in Arabidopsis, we conducted experiments to determine whether TaNRAMP3 can restore the Mn deficiency phenotype in Arabidopsis *nramp1* mutants. We introduced the CDS sequence of *TaNRAMP3* into pCAMBIA-1381 to obtain the complement line *nramp1*/*TaNRAMP3* under the control of the *AtNRAMP1* promoter. Mn deficiency phenotype experiments revealed that *nramp1* mutants displayed a sensitive phenotype under Mn deficiency, characterized by a reduction in root length and fresh weight. However, the *nramp1/TaNRAMP3* transgenic plants were able to compensate for the sensitive phenotype of the *nramp1* mutant, as they did not show any differences in root length or fresh weight compared to the wild-type (WT) plants (Fig. 5A-C). These results indicate that *TaNRAMP3* serves as the orthologous gene of *AtNRAMP1* in *Triticum aestivum*.

To further verify the function of TaNRAMP3 in Arabidopsis, we introduced TaNRAMP3 driven by *CaMV35S* promoter into Arabidopsis WT plants to obtain TaNRAMP3 overexpression lines. The results revealed that WT/*TaNRAMP3* transgenic plants showed a significant tolerance phenotype compared to the WT under Mn-deficiency condition as evidenced by increased root length and fresh weight (Fig. 5D-F). This growth phenotype is also consistent with the phenotype of *AtNRAMP1* overexpressed plants under Mn deficiency condition (Cailliatte et al. 2010). To investigate whether *TaNRAMP3* also alleviate the Fe or Zn deficiency sensitive phenotypes observed in *nramp1* mutants, we performed Fe deficiency and Zn deficiency phenotypes analyses in *nramp1*/TaNRAMP3 plants. The results revealed that *TaNRAMP3* partially rescue for the Fe deficiency and Zn deficiency sensitivities observed in *nramp1* mutants (Supplemental Figure 7), thereby corroborating the findings of the heterologous yeast complementation analysis. These results further suggested that *TaNRAMP3* plays a crucial role in Mn transport under Mn deficiency conditions.

## Discussion

To date, orthologous proteins of AtNRAMP1 have been characterized in various species, such as rice and corn. They are play essential roles in the tolerance to the Mn deficiency condition (Sasaki et al. 2012; Ueno et al. 2015; Guo et al. 2022). Previous research has shown that TpNRAMP3 in dwarf Polish Wheat (*Triticum polonicum* L) possesses Mn, Cd, and Co transport activities, and improve tolerance to Mn toxicity in Arabidopsis (Peng et al. 2018). Nevertheless, the functions of TpNRAMP3 in *Triticum aestivum* and its roles under Mn deficiency were not mentioned (Peng et al. 2018). In this study, we demonstrate that TaNRAMP3 is an essential Mn transporter in *Triticum aestivum*. The Mn deficiency phenotypes of *TaNRAMP3-OE* and *TaNRAMP3-RNAi* plants indicated that TaNRAMP3 plays a crucial role in Mn transport under Mn deficiency condition. The determination of Mn content indicated that the Mn deficiency phenotype of T*aNRAMP3-OE* and *TaNRAMP3-RNAi* may be caused by a decrease in Mn content in the shoots. Research reported that a decrease in Mn content can lead to a decline in photosynthesis, ultimately leading to growth and development limitations and a decrease in yield (Andresen et al. 2018; Li et al. 2019). Therefore, the growth and development phenotypes of *TaNRAMP3-OE* and *TaNRAMP3-RNAi* transgenic lines, such as photosynthesis and fresh weight, may be indirectly caused by differences in Mn content in the plants.

In this study, the function of TaNRAMP3 in influx Mn transport was detected using yeast mutants (*Δsmf1*). TaNRAMP3 was found to rescue the growth inhibition of this yeast mutant under Mn deficiency conditions (Fig. 2). We propose that TaNRAMP3 mediates Mn transport in yeast to maintain intracellular Mn concentration, which is crucial for yeast growth under Mn deficiency condition. Furthermore, as TaNRAMP3 is also localized in the PM of *Triticum aestivum* protoplasts (Fig. 1A-B), it is suggested that TaNRAMP3 may also contribute to Mn transport in plant cells, similar to AtNRAMP1. As a result, we hypothesize that, in addition to Mn^2+^, TaNRAMP3 may be involved in the transport of Fe and Zn like other NRAMP members (Cailliatte et al. 2010; Socha and Guerinot 2014). The findings revealed that TaNRAMP3 can transport Fe and Zn in yeast; however, its transport capacity was weak and not sufficient to fully restore the growth sensitivity of yeast mutants (*Δfet3fet4* and *Δzrt1zrt2*) to the wild-type level (Fig. 2).

Since TaNRAMP3 and AtNRAMP1 have similar functions in Mn transport. We performed qRT-PCR to detect whether the expression pattern of TaNRAMP3 is similar to that of AtNRAMP1, which is primarily expressed in roots (Cailliatte et al. 2010). However, we found that while *TaNRAMP3* is induced by Mn deficiency, its expression level is higher in the leaves, suggesting that TaNRAMP3 may have additional functions beyond those shared with AtNRAMP1. The induction of *TaNRAMP3* by Mn deficiency implies precise transcriptional regulation. In addition, recent studies have shown that NRAMP1 was phosphorylated by the protein kinases CPK21/23 and CIPK23 in Arabidopsis (Fu et al. 2022; Zhang et al. 2023a; Huang 2022), The 24th residue of TaNRAMP3 protein is also serine, which corresponds to the Ser22 of AtNRAMP1, indicating that TaNRAMP3 may also undergo phosphorylated. Therefore, exploring the transcription factors and protein kinases that regulate TaNRAMP3 will be one of our future research.

## Conclusion

In conclusion, we identified TaNRAMP3 as a transporter located in the plasma membrane of *Triticum aestivum*. This transporter plays a crucial role in plant growth and development by mediating the transport of Mn. These insights solve the long-term enigma of how mechanistically initiate their adaptation response to Mn deprivation in wheat and facilitate the cultivation of Mn-deficiency-tolerant crops.

## Materials and methods

### Plant materials and growth conditions

Wild type (WT) plants used in this study were Columbia (Col-0) background in Arabidopsis and the WT were fielder in *Triticum aestivum*. The T-DNA insertion lines *nramp1* (SALK_053236C) were obtained from *Nottingham Arabidopsis Stock Centre* (NASC).

*Arabidopsis* seeds were grown on a nutrient medium consisting of 1% agar (Sigma-Aldrich; A1296; USA), 1% sucrose, and full-strength Hoagland nutrient solution (5 mM KNO_3_, 5 mM Ca(NO_3_)_2_, 2 mM MgSO_4_, 1 mM NH_4_H_2_PO_4_, 20 μM MnSO_4_, 3 μM H_3_BO_3_, 1 μM (NH_4_)_6_Mo_7_O_24_, 0.4 μM ZnSO_4_, 0.2 μM CuSO_4_, 20 μM Fe (III)-EDTA) at pH 5.75 ~ 5.85, and -Mn medium (full strength Hoagland nutrient solution without MnSO_4_), grown on vertical plates at 21 °C for 10 days, 16 h light/8 h dark cycle (Gao et al. 2018). The medium for Fe deficiency and Zn deficiency referred to the previously published methods, and some adjustments were made (changed 1/20 Hoagland to 1 × Hoagland) (Wang et al. 2023; Gao et al. 2018).

For phenotyping assay in *Triticum aestivum*, WT, *TaNRAMP3-OE* and *TaNRAMP3-RNAi* plants were grown hydroponically in Hoagland medium for one week and then transferred to Mn-deficiency medium for another two weeks. All phenotypic experiments were repeated three times (*n* = 15 for each genotype). The root length was measured with ImageJ.

### Plasmid construction

To construct the overexpression vector, the CDS of *TaNRAMP3* were fused to the *pCAMBIA-1300-GFP* vector by homologous recombination (Manishankar et al. 2018), with the GFP tag following the CDS. The *RNAi* vector was constructed by amplifying the 150 bp highly specific fragment of TaNRAMP3 and then linked into *RNAi* vector PC336 (Wang et al. 2022). The constructs were transformed into *Triticum aestivum* to obtain *TaNRAMP3-OE* and *TaNRAMP3-RNAi* transgenic lines.

For genetic complementation analysis in Arabidopsis, the 1300 bp *AtNRAMP1* promoter and 1,647 bp CDS of *TaNRAMP3* were fused to pCAMBIA-1381 vector to obtain the *ProAtNRAMP1*:*TaNRAMP3* vector. The CDS of *TaNRAMP3* were fused to the *pCAMBIA-1307-FLAG* vector by homologous recombination, with the FLAG tag following the CDS.

*Agrobacterium tumefaciens* strain EHA105 were used to transform *Triticum aestivum* or Arabidopsis plants. The primers used for plasmid construction are listed in Table S1.

### Semi-quantitative RT-PCR and quantitative RT-PCR

The RT-PCR was based on the published method (Liu et al. 2023). In brief, Sample total RNA was extracted using an RNA simple total RNA kit (Tiangen, DP419; China), and first-strand cDNA was synthesized from total RNA with the HiScript II Q RT SuperMix for qPCR (+ gDNA wiper) (Vazyme, R223-01; China). The HiScript II 1st Strand cDNA Synthesis Kit (+ gDNA wiper) for Semi-quantitative RT-PCR (Vazyme, R212-01). Quantitative RT-PCR was performed according to the instructions provided for the Real-time PCR instrument (CFX connect, Bio-Rad, USA) using ChamQ SYBR qPCR Master Mix (Vazyme, Q311-02). Statistical differences between the samples were evaluated by ANOVA. The specific primers used are listed in Table S1.

### Subcellular localization of TaNRAMP3

The transformation and expression of mesophyll cell protoplasts were based on the published method (Yoo et al. 2007). In brief, Mesophyll cell protoplasts were prepared by leaf digestion of wild-type plant Fielder *Triticum aestivum* seedlings under dark conditions. TaNRAMP3-GFP fusion constructs were transformed into *Triticum aestivum* mesophyll cell protoplasts by polyethylene glycol induction. The transformed protoplasts were incubated in darkness at 23 °C for 12–15 h, and then 60x objective lens was used (Olympus IX83-FV3000; Japan). The excitation wavelength was 488 nm, and the emission wavelength was 500–530 nm. The ratio of PM to intracellular signal was analyzed by ImageJ.

### Functional analysis in yeast

The functionnal analysis in yeast was based on the published method (Milner et al. 2013). In brief, Yeast vectors expressing TaNRAMP3 and AtNRAMP1 were transformed into yeast strains Δ*fet3fet4*, Δ*smf1* and Δ*zrt1/zrt2* respectively. SD-U liquid medium was used for yeast culture grown to OD = 0.1. Four 10-fold series of diluents were established under sterile conditions. For growth curve determination, the OD600 values of the yeast mutants were recorded every 3 h after 18 h of growth with low Mn (2 mM EGTA) or Fe (20 μM BPDS) or Zn (1 mM EGTA).

### Elemental analysis

Tissues were desorbed by washing with 2 mM CaSO_4_ and 10 mM EDTA for 5 min, followed by a 10 min rinse with ddH_2_O. The samples were dried at 65 °C for one week. For mineralization, plant samples and 5 mL of nitric acid were added to the digestion tube, and digestion was carried out at 120 °C for 5 h. Subsequently, 2 mL of H_2_O_2_ was added to the digestion tube in two portions and the temperature was maintained. Then, the temperature was raised to 160 °C, and maintained until the nitric acid was completely volatilized. Finally, the samples were diluted with ddH_2_O and analyzed by ICP-MS.

### Statistical analysis

Statistical differences were calculated by one-way ANOVA. Different letters indicate means that were statistically different by Tukey’s multiple testing method (*P* < 0.05) for genotypes within a given growth condition (+ Mn or -Mn). For the genotype experiments, confocal microscope experiments, qRT-PCR analysis, and functional analysis in yeast, three independent replicates were performed, a representative image is shown.

### Phylogenetic analysis

The amino acid sequences of NRAMPs in *Triticum aestivum* were obtained from “Ensembl Plants” (http://plants.ensembl.org). Multiple sequence alignments and polygenetic relationships analysis were carried out using the software “DNAMAN6.0” (Huai et al. 2019).

### Supplementary Information

**Additional file 1: Supplemental Figure S1.** Phylogenetic analysis of TaNRAMP3 and selected homologous proteins from other plants. **Supplemental Figure S2.** Schematic view of the structure of TaNRAMP3. **Supplemental Figure S3.** Semiquantitative RT-PCR analysis of the expression level of *TaNRAMP3* and *AtNRAMP1* in yeast mutants. **Supplemental Figure S4.** Relative expression analysis of *TaNRAMP3* under Mn deficiency by qRT-PCR. **Supplemental Figure S5.** The expression analysis of TaNRAMP3-OE and *TaNRAMP3-RNAi* plants. **Supplemental Figure S6.** The expression analyses of *TaNRAMPs* in WT and *TaNRAMP3-RNAi* plants. **Supplemental Figure S7.** Potential functional analysis of AtNRAMP1 and TaNRAMP3 in Arabidopsis. **Table S1.** Primers used in this study.

## Data Availability

All data in this manuscript are available on reasonable request to the corresponding authors.

## Abbreviations

Mn: Manganese
Fe: Iron
Zn: Zinc
Ca: Calcium
PM: Plasma membrane
CDS: Coding sequence
NRAMP: Naturally resistant macrophage protein
EGTA: Ethylene glycol bis (2-aminoethyl ether)-N,N,N’,N’-tetraacetic acid
BPDS: Bathophenanthroline disulfonic acid
ICP-MS: Inductively coupled plasma mass spectrometry
