# Supplementary material for: TaNRAMP3 is essential for manganese transport in Triticum aestivum
Source: Stress Biol. 2023 Sep 22;3(1):41. doi: 10.1007/s44154-023-00120-2 (PMC10516799; doi:10.1007/s44154-023-00120-2)
Supplement: Supplementary file 1 — Additional file 1: Supplemental Figure S1. Phylogenetic analysis of TaNRAMP3 and selected homologous proteins from other plants. Supplemental Figure S2. Schematic view of the structure of TaNRAMP3. Supplemental Figure S3. Semiquantitative RT-PCR analysis of the expression level of TaNRAMP3 and AtNRAMP1 in yeast mutants. Supplemental Figure S4. Relative expression analysis of TaNRAMP3 under Mn deficiency by qRT-PCR. Supplemental Figure S5. The expression analysis of TaNRAMP3-OE and TaNRAMP3-RNAi plants. Supplemental Figure S6. The expression analyses of TaNRAMPs in WT and TaNRAMP3-RNAi plants. Supplemental Figure S7. Potential functional analysis of AtNRAMP1 and TaNRAMP3 in Arabidopsis. Table S1. Primers used in this study. [file 44154_2023_120_MOESM1_ESM.pdf]

TaNRAMP3 is essential for manganese transport in *Triticum aestivum*

Zhangqing Wang#, Yanting Zhang#, Chenyu Cao, Jiaming Liu, Yuan Deng, Zhenqian Zhang* and Cun Wang*.

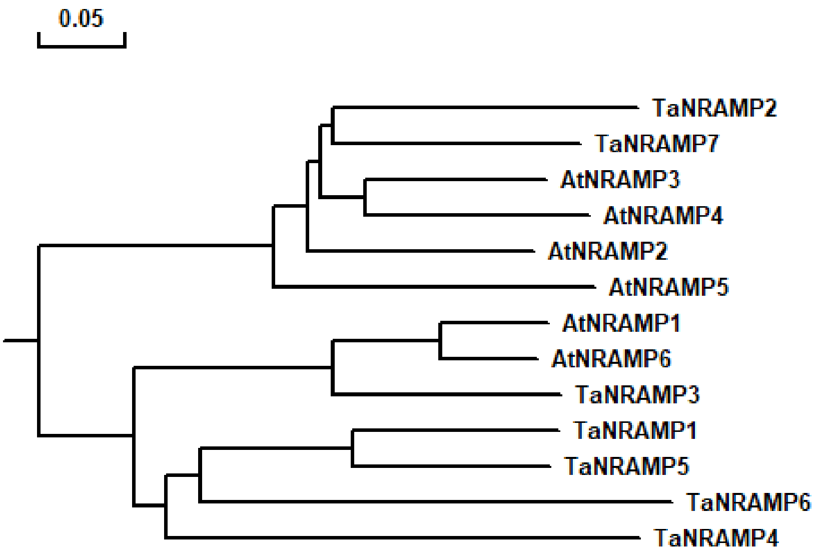

Supplemental Figure S1. Phylogenetic analysis of TaNRAMP3 and selected homologous proteins from other plants.

The phylogenetic tree was constructed based on the maximum likelihood method using DNAMAN6.0. The numbers adjacent to the branch points indicate the percentage of replicates supporting each branch.

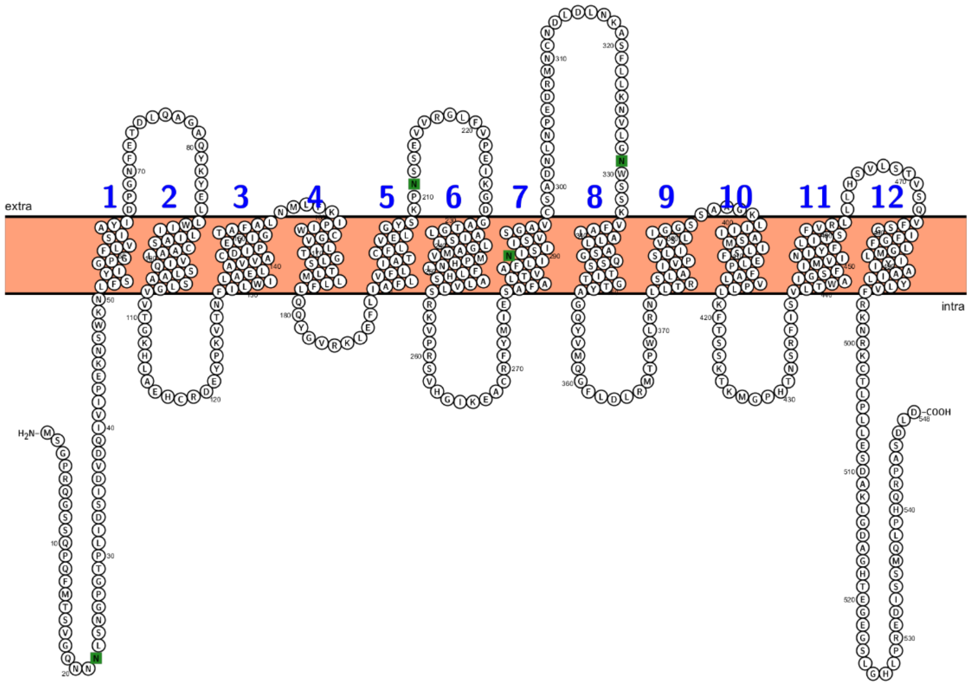

Supplemental Figure S2. Schematic view of the structure of TaNRAMP3.

TaNRAMP3 contains 12 putative transmembrane domains. The N- and C-terminal domains of the protein are located to the cytoplasm. Hypothetical membrane topology of TaNRAMP3 was predicted according to the PROTTER program.

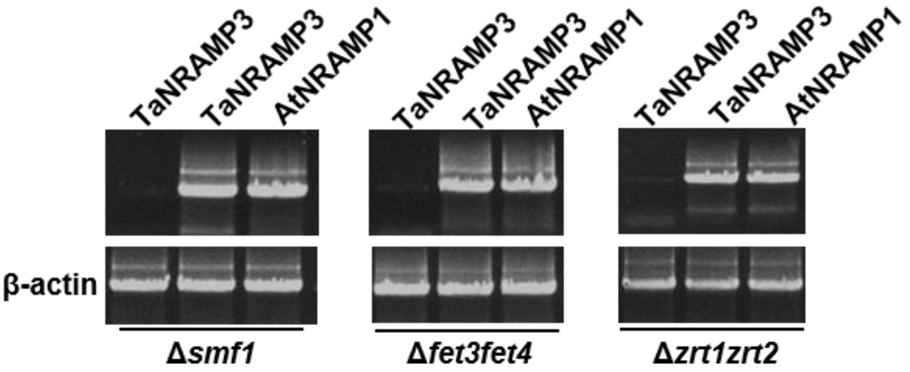

Supplemental Figure S3. Semiquantitative RT-PCR analysis of the expression level of *TaNRAMP3* and *AtNRAMP1* in yeast mutants.

Yeast vectors expressing pYES2, TaNRAMP3 and AtNRAMP1 were transformed into yeast strains *Δfet3fet4*, *Δsmf1* and *Δzrt1/zrt2* respectively. RNA extracted from the yeast mutants grown under ion deficient conditions. *β-actin* is the reference gene.

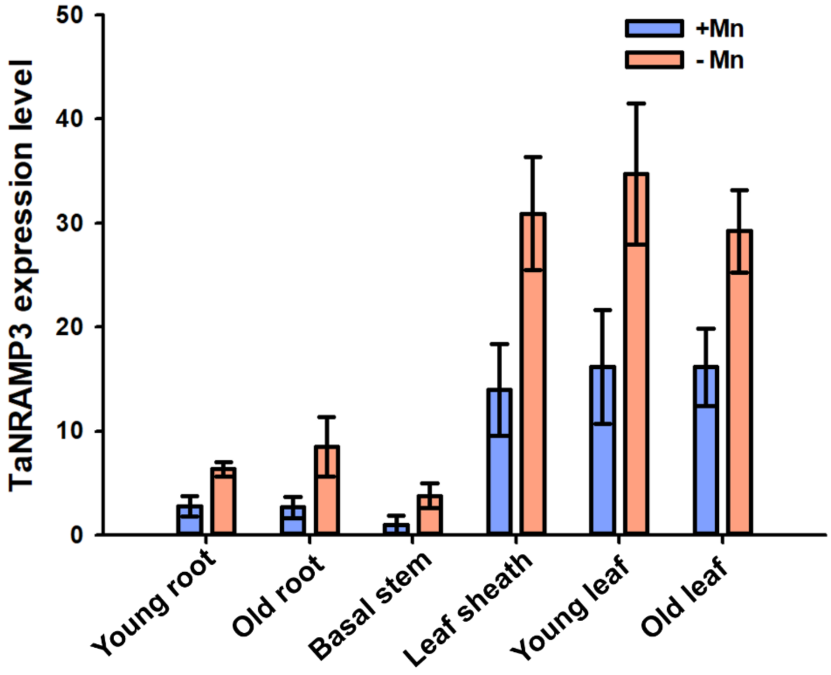

Supplemental Figure S4. Relative expression analysis of *TaNRAMP3* under Mn deficiency by qRT-PCR.

Expression analysis of *TaNRAMP3* under Mn deficiency by qRT-PCR. RNA extracted from the different tissue of WT for the indicated conditions (+Mn or -Mn). *TaACTIN* was used as

an internal control. The results are presented as the mean ± SD (n = 3 biological replicates, 4 technical replicates for each biological replicates). Statistical differences were calculated by one-way ANOVA. Different letters indicate means that were statistically different by Tukey's multiple testing method ($P < 0.05$).

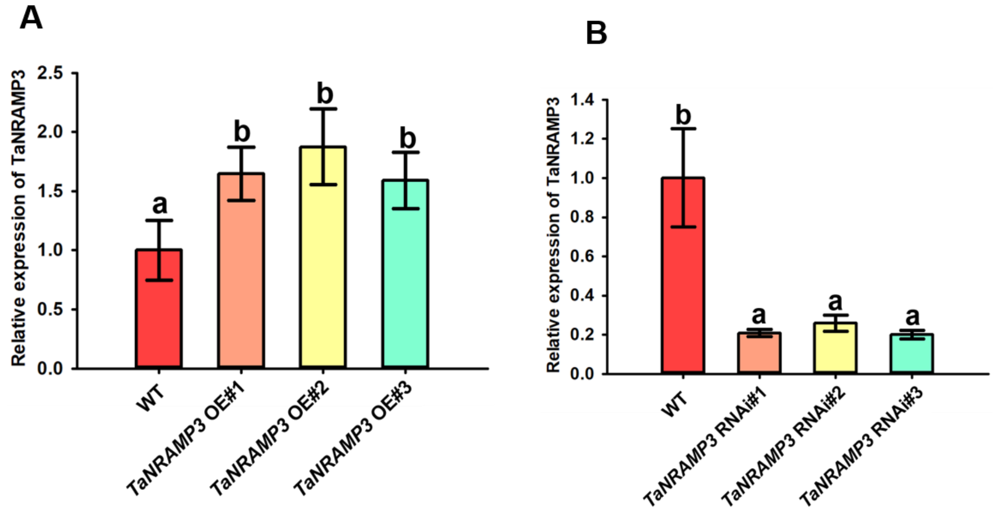

Supplemental Figure S5. The expression analysis of TaNRAMP3-OE and *TaNRAMP3-RNAi* plants.

(A) The relative expression analysis of *TaNRAMP3-OE* plants. *TaACTIN* was used as an internal control. The results are presented as the mean ± SD (n = 3 biological replicates, 4 technical replicates for each biological replicates). Statistical differences were calculated by one-way ANOVA. Different letters indicate means that were statistically different by Tukey's multiple testing method ($P < 0.05$).

(B) The relative expression analysis of *TaNRAMP3-RNAi* plants. *TaACTIN* was used as an internal control. The results are presented as the mean ± SD (n = 3 biological replicates, 4 technical replicates for each biological replicates). Statistical differences were calculated by one-way ANOVA. Different letters indicate means that were statistically different by Tukey's multiple testing method ($P < 0.05$).

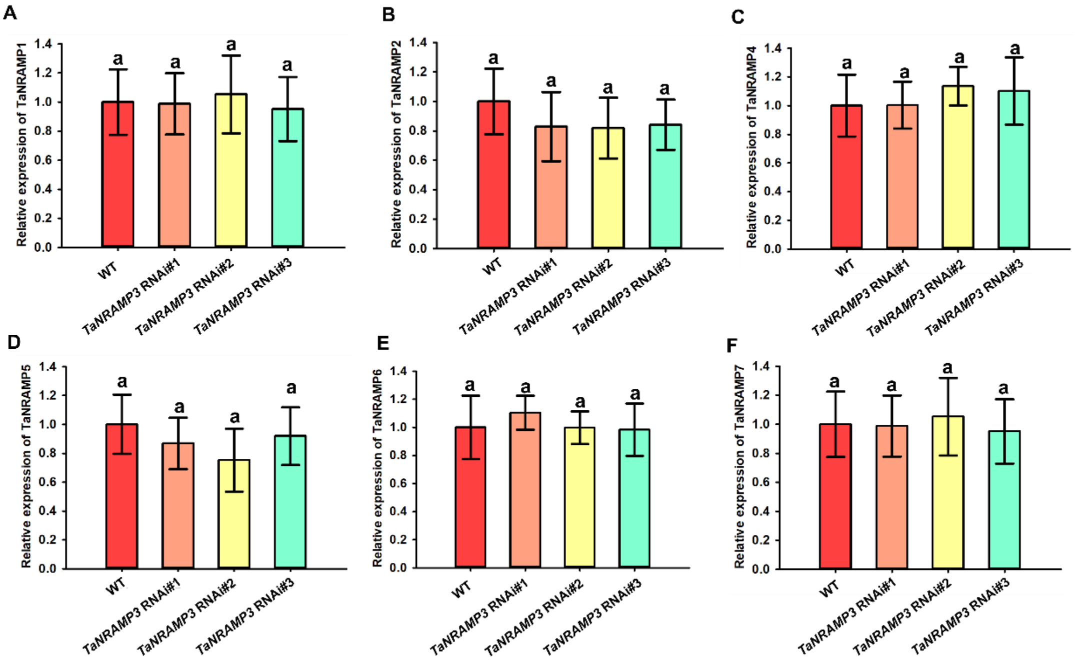

Supplemental Figure S6. The expression analyses of *TaNRAMPs* in WT and *TaNRAMP3-RNAi* plants.

(A) The relative expression analysis of *TaNRAMP1* in WT and *TaNRAMP3-RNAi* plants. *TaACTIN* was used as an internal control. The results are presented as the mean ± SD (n = 3 biological replicates, 4 technical replicates for each biological replicates). Statistical differences were calculated by one-way ANOVA. Different letters indicate means that were statistically different by Tukey's multiple testing method ($P < 0.05$).

(B) The relative expression analysis of *TaNRAMP2* in WT and *TaNRAMP3-RNAi* plants. *TaACTIN* was used as an internal control. The results are presented as the mean ± SD (n = 3 biological replicates, 4 technical replicates for each biological replicates). Statistical differences were calculated by one-way ANOVA. Different letters indicate means that were statistically different by Tukey's multiple testing method ($P < 0.05$).

(C) The relative expression analysis of *TaNRAMP4* in WT and *TaNRAMP3-RNAi* plants. *TaACTIN* was used as an internal control. The results are presented as the mean ± SD (n = 3 biological replicates, 4 technical replicates for each biological replicates). Statistical differences were calculated by one-way ANOVA. Different letters indicate means that were statistically different by Tukey's multiple testing method ($P < 0.05$).

(D) The relative expression analysis of *TaNRAMP5* in WT and *TaNRAMP3-RNAi* plants. *TaACTIN* was used as an internal control. The results are presented as the mean ± SD (n

= 3 biological replicates, 4 technical replicates for each biological replicates). Statistical differences were calculated by one-way ANOVA. Different letters indicate means that were statistically different by Tukey's multiple testing method (P < 0.05).

(E) The relative expression analysis of *TaNRAMP6* in WT and *TaNRAMP3-RNAi* plants. *TaACTIN* was used as an internal control. The results are presented as the mean ± SD (n = 3 biological replicates, 4 technical replicates for each biological replicates). Statistical differences were calculated by one-way ANOVA. Different letters indicate means that were statistically different by Tukey's multiple testing method (P < 0.05).

(F) The relative expression analysis of *TaNRAMP7* in WT and *TaNRAMP3-RNAi* plants. *TaACTIN* was used as an internal control. The results are presented as the mean ± SD (n = 3 biological replicates, 4 technical replicates for each biological replicates). Statistical differences were calculated by one-way ANOVA. Different letters indicate means that were statistically different by Tukey's multiple testing method (P < 0.05).

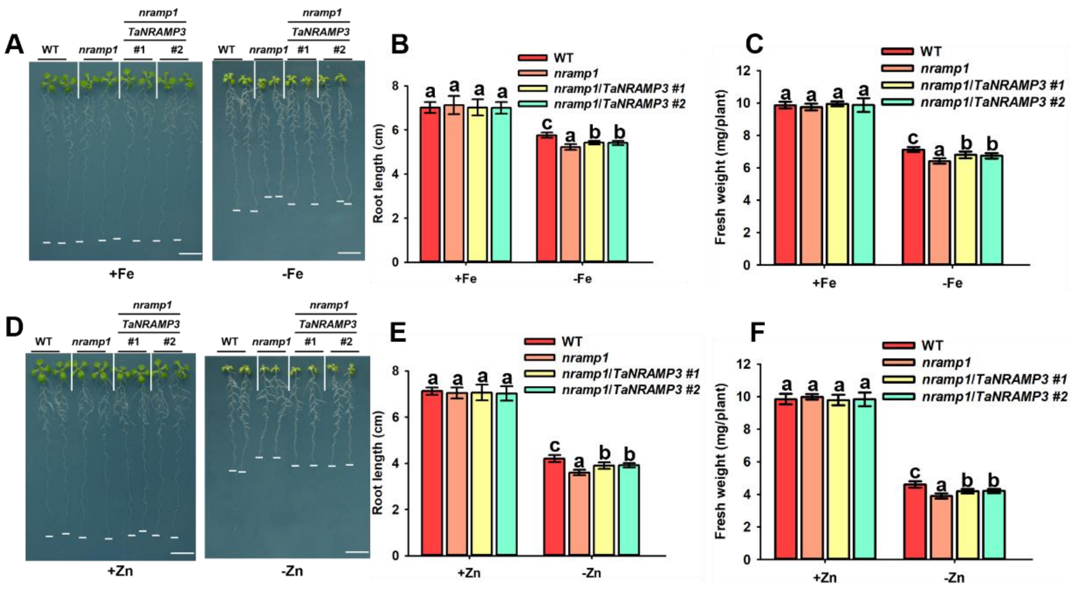

Supplemental Figure S7. Potential functional analysis of AtNRAMP1 and TaNRAMP3 in *Arabidopsis*.

(A) Fe deficiency phenotypes of WT, *nramp1* and *nramp1*/*TaNRAMP3* transgenic plants. Scale bars, 1 cm.

(B) Statistical analysis of root lengths of plants shown in (A). The data are presented as the mean ± SD (n = 15 seedlings for each genotype).

(C) Statistical analysis of fresh weight of plants shown in (A). The data are presented as the mean ± SD (n = 15 seedlings for each genotype).

(D) Zn deficiency phenotypes of WT, *nramp1* and *nramp1*/*TaNRAMP3* transgenic plants. Scale bars, 1 cm.

(E) Statistical analysis of root lengths of plants shown in (D). The data are presented as the mean ± SD (n = 15 seedlings for each genotype).

(F) Statistical analysis of fresh weight of plants shown in (D). The data are presented as the mean ± SD (n = 15 seedlings for each genotype).

Table S1. Primers used in this study.

Name	Sequence (5′—3′)	Application
TaNRAMP3-GFP-F	AGAACACGGGGGACTCTAGAATGAGTGGCCCGAGGCAAGG	Construction of OE plants
TaNRAMP3-GFP-R	ACCATGGTACCCGGGGATCCGTCCAAATCAGACGCAGGGC	
TaNRAMP3-PC336-F	GGCTCCGCGGCCGCCCCCTTATGAGTGGCCCGAGGCAAGG	Construction of RNAi plants
TaNRAMP3-PC336-R	GTGGGCGCGCCGACCCAGCTCTAGTCCAAATCAGACGCAG	
TaSTP6-mCherry-F	AGAACACGGGGGACTCTAGAATGGCCGGCGGTGCGGTTGTAAACA	Localization of TaSTP6 in cell
TaSTP6-mCherry-R	CCCTTGCTCACCATGGTACCCGGGAGCTTGGCAGCCTGGACC	
TaNRAMP3-pYES2-F	GCCGCCAGTGTGCTGGAATTCATGAGTGGCCCGAGGCAAGGCTCAT	Functional analysis in yeast
TaNRAMP3-pYES2-R	TACATGATGCGGCCCTCTAGACTAGTCCAAATCAGACGCAG	
AtNRAMP1-pYES2-F	GCCGCCAGTGTGCTGGAATTCATGGCGGCTACAGGATCTGGACGGT	Functional analysis in yeast
AtNRAMP1-pYES2-R	TACATGATGCGGCCCTCTAGATCAGTCAACATCGGAGGTAGATACT	
NRAMP1-COM-F	CGGCGCGCCGAATTCGTCCAGTAACAATGTACATCATGTG	Cloning of the promoter of AtNRAMP1
NRAMP1-COM-R	AGATTCTAGGGTCGTGGATCGTGTCAAGCTTTAAGAGGAG	
TaACTIN-F	CCGATTGCTTGTTATCTGTT	RT-PCR analysis of

TaACTIN-R	GAGGATGAAGACGAGAGTTT	reference gene
TaNRAMP3-RT-F	CACCAGCAGCAAAACAAAGA	RT-PCR analysis of TaNRAMP3 in plants
TaNRAMP3-RT-R	ATATCAGCATGCCGAGGAAC	
TaNRAMP3-ATG-F	ATGAGTGGCCCGAGGCAAGGCTCAT	Cloning of the CDS of TaNRAMP3
TaNRAMP3-ATG-R	CTAGTCCAAATCAGACGCAG	
β-actin-F	CTGAGAGGGAAATCGTGCGT	RT-PCR analysis of reference gene
β-actin-R	AGGGTGTAAAACGCAGCTCAG	
TaNRAMP1-RT-F	GCATTCGCTTTCAACCTCTT	RT-PCR analysis of TaNRAMP1 in plants
TaNRAMP1-RT-R	TGAATAGCCCCTTCATCACC	
TaNRAMP2-RT-F	CTTCTGATCGGTTTGGTGGT	RT-PCR analysis of TaNRAMP2 in plants
TaNRAMP2-RT-R	GCTTCTTGAACGCGTGATTT	
TaNRAMP4-RT-F	GTTCAGCACCCTCTTGCTTC	RT-PCR analysis of TaNRAMP4 in plants
TaNRAMP4-RT-R	ATGCCCTTGGTCACATCTCT	
TaNRAMP5-RT-F	GTTCATTCCCAAGCTCAAGG	RT-PCR analysis of TaNRAMP5 in plants
TaNRAMP5-RT-R	AAGCCGCTCTCATACAGGAA	
TaNRAMP6-RT-F	GAGCCATGTCAATCCTCCAA	RT-PCR analysis of TaNRAMP6 in plants
TaNRAMP6-RT-R	CATTTATGACGAGCGCAAGA	
TaNRAMP7-RT-F	CGCCTTGGATTGTTTCATTT	RT-PCR analysis of TaNRAMP7 in plants
TaNRAMP7-RT-R	ACAACCCCAACTGCTTGTCT	
